# Preanalytical Considerations of Handling Suspected Creutzfeldt–Jakob Disease Specimens Within the Clinical Pathology Laboratories: A Survey-Based Approach

**DOI:** 10.3390/jcm14010204

**Published:** 2025-01-02

**Authors:** Carla Stephan, Taylor Kalomeris, Yaxin Li, Jeffrey Kubiak, Sabrina Racine-Brzostek, Ivo SahBandar, Zhen Zhao, Melissa M. Cushing, He S. Yang

**Affiliations:** Department of Pathology and Laboratory Medicine, Weill Cornell Medicine, New York, NY 10065, USA; qgh9002@nyp.org (C.S.); tak9098@nyp.org (T.K.); yal4011@med.cornell.edu (Y.L.); jmk9032@med.cornell.edu (J.K.); srb9029@med.cornell.edu (S.R.-B.); ins4002@nyp.org (I.S.); zhz9010@med.cornell.edu (Z.Z.); mec2013@med.cornell.edu (M.M.C.)

**Keywords:** Creutzfeldt–Jakob disease, CJD, prion disease, laboratory handling practice, preanalytical

## Abstract

**Background:** Creutzfeldt–Jakob disease (CJD) is a rare, fatal, and transmissible neurodegenerative disorder caused by prion proteins. Handling specimens from individuals with suspected or confirmed cases presents a safety challenge to hospital workers including clinical laboratory staff. As no national guidelines exist, the clinical pathology laboratory must establish protocols for handling these specimens to ensure sufficient protective measures. This study aims to explore how various medical institutions manage CJD specimens, as a first step toward developing standardized preanalytical protocols for safe specimen handling by health care professionals. **Methods:** An electronic survey was generated and disseminated to diplomats of the American Board of Clinical Chemistry and was posted on the Listserv platform of the American Society for Microbiology and the Artery forum of the Association for Diagnostics and Laboratory Medicine. The survey evaluated various procedures and precautions implemented, the nature of the specimens processed, and whether they are processed in-house or sent to reference laboratories. **Results:** A total of 49 responses were collected. Most respondents (64%) noted their laboratories process specimens with a clinical suspicion of CJD regardless of the level of suspicion, 13% handled specimens only if the degree of suspicion was low, and 16% did not process specimens in-house at all. Among those who process CJD specimens, practices varied greatly, including different levels of precautions, use of biological safety cabinets, aliquoting, disposal, and disinfection procedures. **Conclusions:** A lack of standardization across laboratories exists for the handling of specimens of patients with suspected CJD. This study summarizes the approaches reported by survey respondents, providing a rationale for developing protocols for the safe handling of these specimens and highlighting the need to develop uniform universal standardized processing procedures.

## 1. Introduction

Creutzfeldt–Jakob disease (CJD) is a rapidly progressive neurodegenerative disorder caused by prion proteins. Although rare, this condition is associated with high morbidity and mortality [1]. The mortality rate of CJD has been reported as up to 90%. Clinically, patients initially present with psychiatric symptoms, visual disturbances, or motor dysfunction [2,3]. However, they rapidly develop neurocognitive decline and progression to dementia. Radiologically, hyperintensity in the cerebral cortex and striatum may be seen using diffusion-weighted magnetic resonance imaging (MRI). Microscopic examination of tissue biopsies shows spongiform changes in the gray matter with gliosis, neuronal loss, and major prion protein deposition [1]. The diagnosis of CJD is made based on a combination of clinical symptoms, MRI and electroencephalogram findings, and laboratory testing. In terms of laboratory testing, the presence of protein 14-3-3 in the cerebrospinal fluid (CSF) is a useful, yet non-specific, marker for the diagnosis of CJD [4]. Real-time quaking-induced conversion (RT-QuiC) assay of the CSF is another laboratory test that has been shown to have a sensitivity of 92% and a specificity of 100% [5]. In patients with suspected CJD, additional laboratory testing is usually conducted to exclude other treatable conditions such as neoplastic, inflammatory, or infectious conditions. Thus, the laboratory may receive CSF or other body fluids for routine testing, such as cell counts, metabolic analysis, cytology, flow cytometry, and microbiology cultures, to investigate other processes.

Most cases are sporadic, however, CJD can also be inherited genetically. Rarely, cases can result from direct contact with contaminated tissue. Within the medical field, iatrogenic transmission of the CJD prion has recently been reported in approximately 500 cases worldwide; however, all of these cases occurred in the surgical setting prior to the implementation of routine sterilization and decontamination procedures [6].

To date, no iatrogenic CJD transmission has been reported in the clinical laboratory setting; however, body fluids from individuals with CJD are considered to have infectious potential and thus present a safety challenge for clinical laboratories. Therefore, proper handling of specimens from patients with suspected or confirmed CJD in the clinical laboratory is of the utmost importance to ensure the health and safety of laboratory personnel.

The biological source and the degree of clinical suspicion of CJD are important factors to consider when handling these specimens. Some laboratories across the United States do not test specimens of patients with suspected CJD, and instead send these specimens to reference laboratories. While this strategy has merit, the consequences of delayed turnaround time for the cell counts and metabolic testing must be considered and balanced against the inherent risk of the specimen.

Unlike urine and feces, which have been categorized as having no detectable infectivity, CSF and blood are biological sources that are classified as having low infectivity in experimental models according to the World Health Organization (WHO) [7]. The Centers for Disease Control and Prevention (CDC) offers guidance on infection control procedures for the handling of prion-related specimens [8], and the WHO developed infection control guidelines for transmissible spongiform encephalopathies [7]. However, there are no standardized national guidelines in place for clinical pathology laboratory practices. This study aimed to assess the current preanalytical considerations of specimen handling and processing strategies employed by clinical pathology laboratories for specimens from patients with suspected or confirmed CJD, opening the conversation to standardize protocols for CJD specimen processing.

## 2. Materials and Methods

A survey (Supplementary Materials) to assess practices for handling CJD specimens was generated. This survey was divided into two parts: demographics of the responders and handling practices. The survey had a list of questions in addition to the ability to add comments. The types of specimens received from patients with suspected CJD were assessed, such as blood, CSF, and other fluids. The individual tests performed on these specimens were also evaluated, such as cell counts and differentials, metabolic testing, cultures, etc. Finally, the processing methodologies and the biosafety precautions employed were assessed. The survey was limited to clinical pathology laboratories and was not sent to primary anatomic laboratory associations or listservs. Specifically, the survey was sent to diplomats of the American Board of Clinical Chemistry and was posted on the Listserv platform of the American Society for Microbiology and the Artery forum of the Association for Diagnostics and Laboratory Medicine. The responses remained anonymous. The survey was sent during February and March 2023. Responses were considered incomplete and excluded if less than 10% of the survey was completed. Descriptive statistics were employed to analyze the collected data. Once the data were collected and analyzed, they were shared with the responders.

## 3. Results

A total of 49 responses were collected. Four incomplete responses were received and removed from the analysis. Completed responses were submitted by 37 respondents, while near-complete surveys were tendered by 8 respondents (with a range of 16–90% completion). The characteristics of the responders are provided in Table 1. The responses to the survey are summarized in Table 2.

### 3.1. Demographic Characteristics of the Responders

Most of the respondents were medical directors (31 out of 45, 69%). Other roles included managers (2%), supervisors (2%), and others (27%). Among the 31 medical directors, 8 respondents were CLIA laboratory directors. Clinical specialties varied from clinical microbiology (23 out of 45, 52%), clinical chemistry (17 out of 45, 38%), hematology and coagulation (1 out of 45, 2%), molecular pathology (1 out of 45, 2%), safety (1 out of 45, 2%), administration (1 out of 45, 2%), and general pathology (1 out of 45, 2%). The plurality of respondents came from academic institutions (18 out of 45, 40%), while other affiliations included reference laboratories (5 out of 45, 11%), non-academic health care systems (11 out of 45, 25%), Veteran Affairs (2 out of 45, 4%), international institutions (5 out of 45, 11%), and unknown institution types (4 out of 45, 9%).

The geographical location of the respondents was recorded in 38 out of the 49 responses. The majority of the respondents were practicing in laboratories in the United States (34 out of 38, 89%). The international responses were from Saudi Arabia (1 out of 38, 3%), Singapore (2 out of 38, 5%), and Canada (1 out of 38, 3%). Within the United States, 7 respondents (21%) were from the South, 7 respondents (21%) were from the West, 8 respondents (23%) were from the Northeast, and 12 respondents (35%) were from the Midwest.

### 3.2. In-House Processing of CSF Specimens Collected from Patients with Suspected CJD

In-house processing of CSF specimens with suspicion of CJD was performed by 35 out of the 45 of the respondents (77%). Of those 35, 29 processed the specimen regardless of the level of suspicion, while 6 only processed the specimen if the level of suspicion was low. The 35 responders who processed in-house encompassed academic institutions (43%), reference laboratories (11%), health care systems (29%), international laboratories (9%), Veterans Affairs hospitals (3%), and unknown institution types (5%). Seven (16%) respondents did not process these specimens in-house, while the remaining three (7%) responded with “unknown.” The respondents who did not process specimens in-house sent the specimens to reference laboratories.

With regards to the processing techniques of CSF specimens, 42% (19 out of 45) of respondents noted that these specimens were processed differently from other CSF specimens while 44% (20 out of 45) of respondents noted that these specimens were not processed any differently. Of the 20 institutions that did not process CSF specimens differently, 5 (25%) mentioned that CSF was considered a low-risk specimen, and routine protocols should be followed in their opinion.

Three of the respondents (7%) used disposable manual hemacytometers and 3 respondents (7%) used biosafety cabinets. Some laboratories indicated that they only processed CSF cell counts, while deferring chemistry testing to a reference laboratory. One of the respondents indicated that they avoided using automated instruments. Three respondents stated their laboratories were not informed when CJD was suspected.

University safety precautions were followed in 31 institutions (69%). Additional safety precautions described by these 31 survey respondents in the comments included the use of biosafety cabinets (3 respondents), separate waste bags and disposal (2 respondents), and disposable equipment (2 respondents). Other precautions included the use of double gloves (1 respondent), an N95 mask (1 respondent), and a separate waste bag (1 respondent). The remaining 14 (31%) indicated “unknown”, “other”, or “not applicable” to the safety precautions survey question.

Fifteen (33.3%) respondents noted that no specific decontamination procedures were in place for CJD specimens. However, among the institutions that did report decontamination procedures, practices varied greatly. Practices included the use of 2% bleach, 10% bleach, and alcohol wipes. One of the respondents stated that in the event of cutaneous exposure, the skin would be soaked with 10% bleach for 3–4 min.

### 3.3. In-House Processing of Blood Specimens Collected from Patients with Suspected CJD

A total of 71% (32 out of 45) of respondents stated that blood specimens of patients with suspected CJD are treated routinely, without any special precautions. Three out of forty-five (7%) respondents stated that special precautions are in place for blood specimens. These respondents were from an academic institution (1), a health care system (1), and the last was from an unknown institution type (1). The precautions included sending out blood specimens to reference laboratories and isolating the blood sample from the remaining workflow. The remaining 22% (10 out of 45) of respondents responded with “unknown”.

### 3.4. In-House Processing of Cytology Specimens Collected from Patients with Suspected CJD

Regarding tissue and cytology samples, 32% (14 out of 45) of respondents stated that these samples are processed regardless of clinical suspicion, while 9% (4 out of 45) only process specimens if the suspicion is low. There were 11 out of 45 respondents (24%) that stated that they do not process such specimens and 35% (16 out of 45) of respondents did not provide a response.

The practices of the respondents were varied with respect to the processing of cytology specimens in-house. For example, one respondent noted that cytology specimens received from the nervous system or eye are not processed in-house, while all other sources are processed internally. Three respondents mentioned that cytology specimens are held until CJD testing at a reference laboratory results as negative.

### 3.5. Notification of Suspected CJD Specimens Arriving at the Laboratory

Among 45 respondents, 25 (56%) noted that their laboratories receive special notification in the event of suspected CJD, while 9 (20%) reported that no special notifications are provided. The remaining respondents (24%) responded as “unknown”. Most notifications consisted of the clinical team informing the laboratory upon sending specimens for testing. There were 2 respondents who mentioned the use of an automated notification system when the protein 14-3-3 test is ordered. Another 2 respondents also noted the use of special labeling stickers to signify CJD suspicion.

## 4. Discussion

This study aimed to assess the strategies employed by clinical pathology laboratories for handling specimens of patients with suspected CJD through a survey-based approach. The survey responses revealed considerable variability in practice, underscoring the lack of standardized practice. In the article, we seek to provide a summary of existing relevant guidelines, recent research findings, and the results of this survey, with the goal of fostering a discussion on standardizing protocols for CJD specimen processing.

Mechanistically, the misfolded prion proteins (PrP) are initially deposited in the extracellular spaces of gray matter in the brain [9], as well as the olfactory epithelial mucosa [10]. The misfolded proteins can then travel through the interstitial fluid that drains through perivascular channels into the CSF, ultimately draining into cervical lymph nodes and to blood. Hence, solid tissue from the brain has the highest concentration of PrP, while the relative concentration diminishes as the abnormal protein flows from the initial source of deposition to the CSF and then to the blood. Infective concentrations from gray matter are orders of magnitude greater than olfactory mucosa, which is in turn orders of magnitude higher than the CSF [11].

Various animal models have been studied to ascertain the low infectious risk of CSF and blood. Inoculation of humanized mice with ~50 μL of CJD-positive CSF did not demonstrate any infectivity. However, other animal models have shown some infectious potential of CJD-positive CSF, typically occurring in the setting of potential CSF contamination by brain tissues, or with atypical pathology non-diagnostic of bovine spongiform encephalopathy [11]. In a mouse experiment, the infectious doses in whole blood and plasma appear to be on the order of 2–4 infective doses per mL, which is the equivalent infectious dose to 0.3–0.5 μg of brain tissue [12]. While such a dose may pose a risk with large volume inoculation in the setting of blood transfusions, this dose may be considered negligible to laboratory workers with accidental exposure [13]. There have been no confirmed cases of occupational CJD transmission to clinical pathology laboratory personnel through blood [7].

Equally important is the route of exposure. Cutaneous exposure through intact skin or mucous membranes (except ocular) has been reported to be of negligible risk. The greatest risk is through the central nervous system or ocular exposure. Working in a clinical laboratory, the greatest potential risk is through direct inoculation through needle-stick injuries or through splashing with ocular exposure [14]. Ocular exposure can be greatly reduced through the use of protective eyewear. There have been no confirmed cases of occupational CJD transmission to clinical pathology laboratory personnel through CSF exposure. However, there has been a reported fatal exposure in a prion research laboratory through a forceps prick contaminated by murine samples (frozen sections of brains of transgenic mice that overexpressed the human prion protein) [15].

In addition to the type of specimens being handled, the degree of clinical suspicion of CJD is another factor that can influence practices. Based on the survey results, practices within a single laboratory varied according to the degree of clinical suspicion. Pre-analytical factors such as the clinical presentation of the patient, the history of exposure, MRI findings, and electroencephalogram results all influence the degree of clinical suspicion. Although the degree of suspicion may influence practices in some laboratories, some respondents mentioned that they are never notified of suspected CJD cases and handle all cases in the same way, regardless of the degree of suspicion. Moreover, in some peculiar cases, such as the Heidenhain variant or the Brownell–Oppenheimer variant of sporadic CJD [2,3], the initial suspicion may be very different, and the laboratory may not be aware that the specimens are from suspected CJD patients and may not take any precautions for the specimens that were already collected earlier.

A prior study in 2012 used a cross-sectional web-based listserv to examine 426 Canadian laboratory workers’ practices, knowledge, and risk of perception when handling prion-related specimens [16]. Their results showed that of the 37% of respondents who reported processing of prion-associated specimens, there was a high perception of risk among laboratory workers and only one-third of the respondents believed they were adequately trained. Importantly, the authors reported that over 80% of respondents would feel more comfortable if national guidelines existed and were implemented in their laboratory. Although our survey did not evaluate risk perception on handling specimens with suspected CJD, many respondents noted that these specimens are of low infective potential.

Based on our survey results, only 7% of our respondents use biosafety cabinets when handling specimens from patients with suspected CJD. The need for biosafety cabinets has not been established or recommended. The WHO does not specify that a biosafety cabinet should be used; however, it recommends that work surfaces should be covered with disposable cover sheets for manual work for low- and high-infectivity specimens [8]. In 1999, the WHO recommended avoidance of automated equipment when processing CSF of patients with suspected CJD and encouraged the use of disposable equipment whenever possible. For example, the use of disposable manual hemocytometers was recommended when performing cell counts on CSF. However, these recommendations have not been updated in 25 years.

Studies have assessed numerous disinfection and sterilization techniques. Most conventional methods have been shown to be insufficient in decontaminating prions. The effectiveness of decontamination procedures is influenced by various factors including the chemical used, the concentration of the chemical, the prion concentration, and the exposure time. Notably, a 1 M sodium hydroxide (NaOH) solution and chlorine at various concentrations have been shown to inactivate prions. In contrast, other chemicals, such as 3% hydrogen peroxide, 2% iodine, 100% alcohol, and 3.7% formaldehyde, have proven insufficient for this purpose [14].

The CDC and WHO recommend discarding contaminated equipment and materials, followed by their destruction through incineration. For instruments that cannot be discarded or in the event of a contaminated surface, it is recommended to flood the surface or instrument with 2 M NaOH for one hour, followed by wiping and rinsing with water. The same procedure applies to heat-sensitive instruments. For heat-resistant instruments, a combination of chemical treatment and autoclaving is recommended for effective decontamination.

This study has limitations. The survey was primarily distributed to laboratory professionals in clinical chemistry and clinical microbiology, and did not reach those in anatomical pathology. As a result, some respondents may not be familiar with procedures for handling flow cytometry or tissue samples. The survey did not collect enough responses regarding the handling of cytology and tissue specimens. Additionally, the number of survey respondents was limited. A larger, more comprehensive survey is needed to gain a broader understanding of the perspectives of the entire pathology and laboratory medicine community on CJD specimen handling.

## 5. Conclusions

This survey examined the various strategies and practices employed by clinical pathology laboratories in managing specimens from patients with suspected Creutzfeldt–Jakob disease. Responses were highly variable, ranging from processing cerebrospinal fluid and blood samples without additional precautions to implementing specialized safety measures, or even sending all specimens to reference laboratories for testing. We recommend the establishment of a national committee to review existing evidence and develop an evidence-based consensus guideline for the laboratory handling of CJD specimens. Further research is needed to investigate the infectivity of different specimen types. Establishing standardized national guidelines for CJD specimen management would not only address concerns related to these high-risk specimens but also enable laboratory staff to handle them with greater efficiency, safety, and confidence.

## Figures and Tables

**Table 1:** Demographic characteristics of the responders.

	*n* (%)
*Hospital Type*	
Academic	18 (40%)
Health Care System	11 (25%)
Reference Laboratory	5 (11%)
International	5 (11%)
Veterans Affairs	2 (4%)
N/A	4 (9%)
*Occupation of survey respondent*	
Medical Director	31 (69%)
Manager	1 (2%)
Supervisor	1 (2%)
Other	12 (27%)
*Laboratory Type*	
Clinical Chemistry	17 (38%)
Clinical Microbiology	23 (52%)
Hematology/Coagulation	1 (2%)
Molecular	1 (2%)
Safety	1 (2%)
Administration	1 (2%)
General Pathology	1 (2%)

CLIA = Clinical Laboratory Improvement Amendments of 1988, N/A = not applicable.

**Table 2:** The questions and responses to the survey.

Question	Responses *n* (%)
*Does your laboratory process and perform testing on CSF specimens collected from patients with suspected Creutzfeldt–Jakob Disease (CJD) in-house?*	
Yes, regardless of level	29 (64%)
Yes, but only low suspicion	6 (13%)
No	7 (16%)
Unknown/Not available	3 (7%)
*If processed and tested in-house, are CSF specimens collected from patients with suspected CJD processed differently from CSF specimens collected from other patients?*	
Yes	19 (42%)
No	20 (44%)
Not applicable	6 (14%)
*If your laboratory performs any testing on CSF specimens collected from patients with suspected CJD in-house, what biosafety procedures do you employ?*	
Universal precautions with no additional safety precautions	31 (69%)
Other	5 (11%)
Not applicable	3 (7%)
Unknown	6 (13%)
*Does your laboratory have any special testing procedures for blood specimens collected from patients with suspected CJD in-house?*	
No, treated routinely	32 (71%)
Yes	3 (7%)
Unknown	10 (22%)
*Does your laboratory process and perform testing on tissue or cytology specimens collected from patients with suspected CJD?*	
Yes, regardless of level of suspicion	14 (32%)
Yes, but only low suspicion	4 (9%)
No	11 (24%)
Not applicable	16 (35%)
*If testing is performed in your laboratory for tissue and/or cytology specimens from patients with suspected CJD, do you employ special procedures or take special precautions for handling?*	
Yes	13 (29%)
No	6 (13%)
Not applicable	8 (18%)
Unknown	18 (40%)
*Do your laboratories receive special notification and/or have dedicated/special procedures for notification from the clinical teams regarding specimens from patients being tested for CJD?*	
Yes	25 (56%)
No	9 (20%)
Unknown	11 (24%)

## Data Availability

A full list of the survey questions is included in the Supplementary Materials.

## Supplementary Materials

The following supporting information can be downloaded at: https://www.mdpi.com/article/10.3390/jcm14010204/s1.
