# Supplementary material for: Preanalytical Considerations of Handling Suspected Creutzfeldt–Jakob Disease Specimens Within the Clinical Pathology Laboratories: A Survey-Based Approach
Source: J Clin Med. 2025 Jan 2;14(1):204. doi: 10.3390/jcm14010204 (PMC11722287; doi:10.3390/jcm14010204)
Supplement: Supplementary file 1 [file jcm-14-00204-s001.zip › jcm-3370383-supplementary.pdf]

1. Name of hospital/institution (note: survey results will be analyzed anonymously)

2. What is your role in the laboratory?
   A. Medical director
   B. Manager
   C. Supervisor
   D. Other, please specify
   E. CLIA lab director

3. What is your specialty?
   A. Clinical Chemistry
   B. Clinical Microbiology
   C. Hematopathology
   D. Hematology/Coagulation
   E. Other please specify

4. Does your laboratory process and perform testing on CSF specimens collected from patients with suspected Creutzfeldt-Jakob disease (CJD) in-house?

   A. Yes, but testing is only performed in house if there is a low level of suspicion for CJD
   B. No
   C. Unknown
   D. Yes, testing is performed in house regardless of the level of suspicion

   Explain/Comment:

5. If processed and tested in-house, are CSF specimens collected from patients with suspected CJD processed differently from CSF specimens collected from other patients? Please provide additional information to explain the differences.

   A. Yes
   B. No
   C. Unknown

   Explain/Comment:

6. When CJD is on the differential, where are the following tests performed? Please check in house or note which reference lab as appropriate.

	In-house	Reference Lab	Unknown
**CSF total protein**			
**CSF glucose**			
**CSF cell count**			
**CSF Differential**			

**CSF cultures**			
**infectious diseases nucleic acid amplification tests (e.g., meningitis/encephalitis panel) on CSF**			
**CSF cryptococcal antigen**			
**CSF Cytology**			
**CSF flow cytometry**			

Explain/comment:

7. If your laboratory performs any testing on CSF specimens collected from patients with suspected Creutzfeldt-Jakob disease (CJD) in-house, what biosafety procedures do you employ?

Please provide additional information to complement your answer.

A. Universal precaution with no additional safety precautions
B. Other, specify
C. Not applicable
D. Unknown

Explain/comment:

8. If your lab performs testing on a CSF specimen and CJD is confirmed later, please explain your process for subsequent decontamination (if any) of laboratory instruments:

Explain/comment:

9. Does your laboratory have any special testing procedures for ***<u>blood</u>*** specimens collected from patients with suspected Creutzfeldt-Jakob disease (CJD) in-house?

A. No, they treated as routine specimens?
B. Yes, please specify
C. Unknown

Explain/comment:

10. Does your laboratory process and perform testing on tissue or cytology specimens collected from patients with suspected CJD?

A. Yes, testing is performed in house regardless of level of suspicion
B. Yes, but testing is only performed in house if there is a low level of suspicion for CJD

C. No
D. Unknown

Explain/comment:

11. If testing is performed in your laboratory for tissue and/or cytology specimens from patients with suspected CJD, do you employ special procedures or take special precautions for handling? Please explain if yes.

A. Yes (please explain)
B. No
C. Not applicable
D. Unknown

12. Do your laboratories receive special notification and/or have dedicated/special procedures for notification from the clinical teams regarding specimens from patients being tested for CJD?

A. Yes
B. No
C. Unknown

Explain/comment:
